# Successful five-item triage for the broad spectrum of mental disorders in pregnancy – a validation study

**DOI:** 10.1186/s12884-015-0480-9

**Published:** 2015-02-28

**Authors:** Chantal Quispel, Tom AJ Schneider, Witte JG Hoogendijk, Gouke J Bonsel, Mijke P Lambregtse-van den Berg

**Affiliations:** Department of Psychiatry, Erasmus MC, University Medical Center Rotterdam, Rotterdam, the Netherlands; Department of Obstetrics and Gynaecology, Division of Obstetrics & Prenatal Medicine, Erasmus MC, University Medical Center Rotterdam, Rotterdam, the Netherlands; Department of Public Health, Erasmus MC, University Medical Center Rotterdam, Rotterdam, the Netherlands; Department of Child and Adolescent Psychiatry, Erasmus MC, University Medical Center Rotterdam, Rotterdam, the Netherlands

**Keywords:** Mental disorders, Personality disorders, Pregnancy, Psychosocial problems, Triage, Validation

## Abstract

**Background:**

Mental disorders are prevalent during pregnancy, affecting 10% of women worldwide. To improve triage of a broad spectrum of mental disorders, we investigated the decision impact validity of: 1) a short set of currently used psychiatric triage items, 2) this set with the inclusion of some more specific psychiatric items (intermediate set), 3) this new set with the addition of the 10-item Edinburgh Depression Scale (extended set), and 4) the final set with the addition of common psychosocial co-predictors (comprehensive set).

**Methods:**

This was a validation study including 330 urban pregnant women. Women completed a questionnaire including 20 psychiatric and 10 psychosocial items. Psychiatric diagnosis (gold standard) was obtained through Structured Clinical Interviews of DSM-IV axis I and II disorders (SCID-I and II). The outcome measure of our analysis was presence (yes/no) of any current mental disorder.

The performance of the short, intermediate, extended, and comprehensive triage models was evaluated by multiple logistic regression analysis, by analysis of the area under the ROC curve (AUC) and through associated performance measures, including, for example, sensitivity, specificity and the number of missed cases.

**Results:**

Diagnostic performance of the short triage model (1) was acceptable (Nagelkerke's R^2^=0.276, AUC=0.740, 48 out of 131 cases were missed). The intermediate model (2) performed better (R^2^=0.547, AUC=0.883, 22 cases were missed) including the five items: ever experienced a traumatic event, ever had feelings of a depressed mood, ever had a panic attack, current psychiatric symptoms and current severe depressive or anxious symptoms. Addition of the 10-item Edinburgh Depression Scale or the three psychosocial items unplanned pregnancy, alcohol consumption and sexual/physical abuse (models 3 and 4) further increased R^2^ and AUC (>0.900), with 23 cases missed. Missed cases included pregnant women with a current eating disorder, psychotic disorder and the first onset of anxiety disorders.

**Conclusions:**

For a valid detection of the full spectrum of common mental disorders during pregnancy, at least the intermediate set of five psychiatric items should be implemented in routine obstetric care. For a brief yet comprehensive triage, three high impact psychosocial items should be added as independent contributors.

**Electronic supplementary material:**

The online version of this article (doi:10.1186/s12884-015-0480-9) contains supplementary material, which is available to authorized users.

## Background

Pregnancy and childbirth are sensitive periods in which mental disorders can arise or relapse [1]. The occurrence of mental disorders during pregnancy varies across studies. Prevalence rates of 13% for major depressive disorder, 1% for bipolar mood disorders, 1% for substance use disorder, 2% for panic disorders, 4% for post-traumatic stress disorder, 9% for generalized anxiety disorder, 1% for obsessive-compulsive disorder, 4% for eating disorder, and 6% for personality disorders have been reported in several recent studies from Western countries, mainly using self-report questionnaires [1,2]. Despite the high prevalence and subsequent short- and long-term adverse health outcomes for both mother and child [3-5], mental health is not always part of routine prenatal care [6]. Consequently, detection and treatment rates of pregnant women with mental disorders are low. Reasons include professional’s lack of expertise and education, reluctance to take responsibility for case management, and avoidance of stigmatisation of both women and professionals. If not asked specifically, women are not inclined to report mental health symptoms spontaneously [6,7].

In the city of Rotterdam, obstetricians and psychiatrists agree on a structured triage for mental disorders during pregnancy. Besides the general history, pregnant women with mental disorders are guided to psychiatric consultation on behalf of a short set of three psychiatric triage items: previous hospital admission of the woman for psychiatric disorder, previous hospital admission of a first-degree relative for psychiatric disorder, or previous psychotropic medication use. This selection was based on prior studies that consistently showed that psychiatric history is the strongest predictor for future psychiatric disorders [1,8]. For triage purposes, we aim at the most serious disorders, for which psychiatric admission or medication use is needed. We additionally ask for hospital admission of a first-degree relative as a general marker for increased vulnerability for psychiatric disorders, and more specifically because of the strongly increased risk for postpartum psychosis in women with a first-degree relative suffering from bipolar disorder [9].

To further facilitate obstetrical professionals in the triage of mental disorders during pregnancy, several screening instruments have been developed worldwide. Most instruments show limitations in diagnostic coverage. First, most instruments - such as the commonly used Edinburgh Depression Scale - only focus on the most common mental disorders such as depression and anxiety [10-14]. Second, personality disorders are not included despite the fact that these disorders are prevalent during pregnancy and are known to worsen health outcomes and complicate treatment in case of comorbid conditions [15]. Third, comorbid conditions such as insufficient social support and substance use are claimed to be strong independent co-predictors for mental disorders [16,17] but are rarely incorporated in screening or triage.

A trade-off exists between a) the comprehensiveness of instruments, including mental disorders, and comorbid psychosocial stressors or substance abuse, and b) brevity, including a limited number of items, but with a rather high correlation to the broad spectrum of mental disorders.

To improve the triage of the broad spectrum of DSM-IV axis I and II disorders during pregnancy, this paper investigated the decision impact validity of: 1) the currently used short set of three psychiatric items, 2) this set after addition of seven specific psychiatric items (intermediate set), 3) this set after the further addition of the 10-item Edinburgh Depression Scale (extended set), and 4) the final addition of common psychosocial co-predictors (comprehensive set). We hypothesized that the addition of at least some specific psychiatric screening items would be superior to the currently used short set of screen items in order to predict psychiatric disorders during pregnancy.

## Methods

### Procedure

After complete description of the study to the subjects, written informed consent was obtained. Data were generated by the self-reported Mind2Care screen-and-advice instrument (formerly known as GyPsy instrument) [18] and a set of seven additional psychiatric self-reported screening items. Mind2Care was primarily developed by the Erasmus Medical Center as a tool for screening and subsequent treatment allocation for psychiatric and psychosocial risk factors during pregnancy. Mind2Care includes a short set of currently used psychiatric triage items (previous hospital admission of the woman for mental disorder, previous hospital admission of first-degree relative for mental disorder, previous psychotropic medication use). Additional psychiatric triage items were suggested by clinicians who screened more than 2300 pregnant women for mental disorders. Based on these suggestions and comprehensive literature sources [1,16,19-23] seven additional items were selected to be validated in combination with the short set of items (which together form the intermediate set): previous professional psychiatric treatment, previous traumatic experience, previous feelings of a depressed mood, panic attack, current psychiatric symptoms, current severe depressive or anxious symptoms, current severe fear of childbirth (see Additional file 1). Mind2Care also included the 10-item Edinburgh Depression Scale (EDS) [24] (if added: the extended set), and ten psychosocial stressors, including life events and substance use (unplanned pregnancy, unwanted pregnancy, insufficient social support, relational problems, financial debts, unstable housing, sexual or physical abuse, smoking, alcohol consumption and illicit drug use) (comprehensive set), and a set of characteristics (maternal age, ethnicity, socioeconomic status, educational level, marital status, gestational age, gravidity, and parity) [18]. All women filled out the Mind2Care and the seven additional psychiatric triage items independently.

### Outcome

Outcome measure was defined as any current mental disorder diagnosed using the Structured Clinical Interview for the DSM-IV axis I and axis II disorders (SCID-I and SCID-II) [20,21]. SCID-I assesses major mental disorders of the DSM-IV axis I divided into seven primary classes: mood, psychotic, substance, anxiety, somatic symptom disorder, eating, and adjustment disorders. SCID-II assesses the eleven DSM-IV personality disorders divided into cluster A, B and C personality disorders. SCID responses include a 3-point rating with 1 indicating ‘no’ , 2 indicating ‘yes, sub-threshold’ , and 3 indicating ‘yes, supra-threshold’. Psychiatric diagnoses are based on the underlying SCID algorithm and scoring system. The 18 SCID classifications presented here cover all of the information available from SCID-I and II.

SCID interviews were conducted by a formally trained, certificated researcher (C.Q.) in private rooms at the outpatient departments of the participating hospitals or at the women’s homes. The interviewer was blinded to all previous reported data. Due to the design of the SCID, the interviews lasted from 15 minutes for women without any mental disorder to 3 hours for women with mental disorders. Outcomes measures were equal for both groups.

### Participants

To include a reliable heterogeneous sample of pregnant women with psychiatric disorders and women without psychiatric disorders, we first approached a preselected sample of 188 pregnant women at high risk for mental disorders from a tertiary hospital. This sample included pregnant women who were referred to this tertiary hospital for psychiatric symptoms by their general practitioner or midwife, and women with a history of psychiatric symptoms, from September 2011 to July 2013. From May 2012 to July 2012 we approached an unselected sample of 512 pregnant women at low risk for mental disorders from a midwifery practice and an obstetric outpatient department of a general hospital to participate in this study. All practices were located in Rotterdam, the second largest city in the Netherlands.

**Figure 1:**
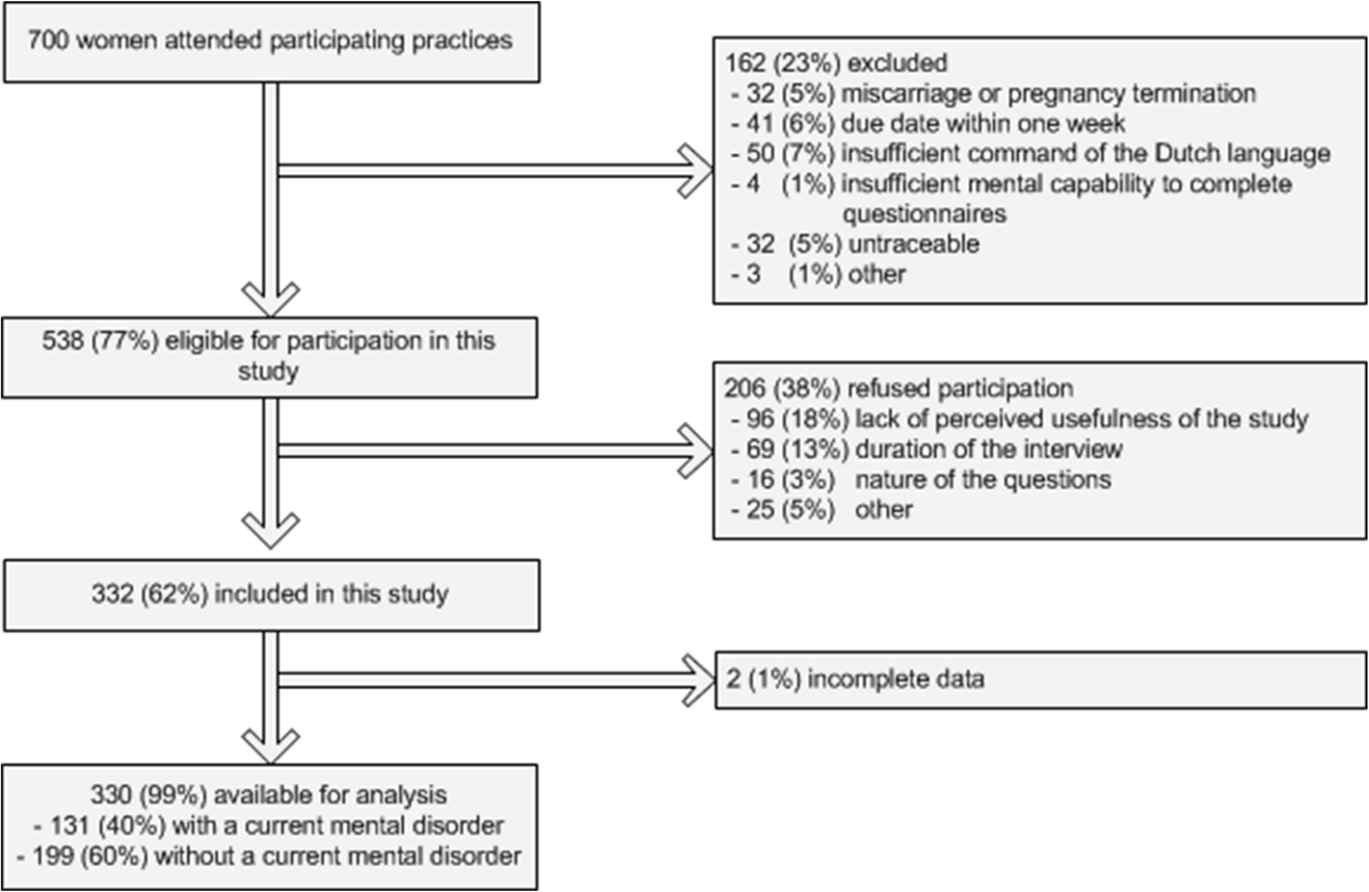
**Study profile.**

Exclusion criteria included having a miscarriage at the time of screening, being non-Dutch speaking, and having insufficient mental capability to complete the Mind2Care independently. In total, 538 pregnant women fulfilled the inclusion criteria. As 206 women refused participation and two women had too many missing data points, 330 women were included (Figure 1). Women did not receive a reward for participation. An a priori sample size calculation defined that a sample of at least 120 women with a mental disorder was needed to conduct statistical analysis with a power of 0.8 and a 95% confidence interval.

This study was approved by the institutional review board of the Erasmus University Medical Center (MEC-2011-101).

### Statistical methods

Frequency tables, chi-squared tests and Mann–Whitney U tests were used to describe and compare the study groups. The proportion of characteristics, psychiatric and psychosocial triage items according to the presence of mental disorders were examined for comparative reasons only. To investigate the model fitness of the short, intermediate, extended, and comprehensive set of triage items, first multiple logistic regression modelling was applied with current psychiatric disorder (yes/no) as a binary outcome measure. The short set of three clinically-used psychiatric items were entered into model 1. Then the performance of the intermediate set of psychiatric items in model 2 was tested, applying stepwise backwards regression. The Edinburgh Depression Scale was added in model 3 (extended set). Finally we tested the additive value of psychosocial items, including life events and substance use (model 4, comprehensive set of items). At this stage, we deliberately did not adjust for women’s characteristics, as we focused on the performance of different sets of items in a heterogeneous group of pregnant women. Women’s characteristics were, however, entered in a fifth model to explore the sensitivity of the triage models. Sensitivity was explored to ensure validity under routine care conditions, without the exceptions of any subgroup, for example lowly educated women. Model fitting was assessed by chi-squared and Nagelkerke's R-squared statistics, and Hosmer Lemeshow statistics reflecting classification power of a group of variables.

Discriminant validity of the four triage models was examined with the area under the Receiver Operator Characteristics (ROC) Curve. The Area Under the Curve (AUC) was calculated for all four models, with a value of 0.70 representing acceptable discrimination, 0.80 representing excellent discrimination, and 0.90 representing outstanding discrimination. Test performances in terms of sensitivity, specificity, positive predictive value (PPV) and negative predictive value (NPV) were calculated using a cut-off point of 0.4. The cut-off of 0.4 was chosen as it reflects the proportion of mental disorders in our sample.

All analyses were performed using the Statistical Package for Social Science, version 20.0.

## Results

Table 1 reports the proportion of current mental disorders of the DSM-IV axis I and axis II in the study groups. Forty per cent of women had a current mental disorder, with mood, anxiety and personality disorders being the most prevalent.

**Table 1:** **Psychiatric diagnosis of study participants established by SCID I and SCID II** ^**1**^ **(n = 330)**

	**Participants (n = 330)**	
***Psychiatric diagnosis***	***n***	***(%)***
Current mental disorder (axis I or II)	131	(40)^2^
Current mental disorder on DSM-IV axis I	110	(33)
Mood disorder	57	(17)
Bipolar disorder type I or II	1	(0)
Major depressive disorder	54	(16)
Dysthymic disorder	2	(1)
Psychotic disorder	8	(2)
Substance related disorder	7	(2)
Anxiety disorder	71	(22)
Panic disorder	43	(13)
Phobias	13	(4)
Obsessive-compulsive disorder	5	(2)
Post-traumatic stress disorder	20	(6)
Generalized anxiety disorder	14	(4)
Somatoform disorder	0	(0)
Eating disorder	5	(2)
Adjustment disorder	4	(1)
Current mental disorder on DSM-IV axis II	56	(17)
Cluster A personality disorder	0	(0)
Cluster B personality disorder	33	(10)
Cluster C personality disorder	30	(9)

^1^Diagnosis based on Structured Clinicial Interview of DSM-IV disorders axis I and II (SCID I and SCID II).

^2^Including 44 women of the unselected cohort (low a priori risk for mental disorders) and 87 women of the preselected cohort (high a priori risk for mental disorders).

Table 2 shows the characteristics and triage items for the total group of women, and separately for women with and women without a current mental disorder. Women with mental disorders were more often of non-Western ethnicity (44% versus 32%), less often highly educated (27% versus 41%), and more often single as compared to women without mental disorders (8% versus 3%, all p < 0.05). All psychosocial and psychiatric triage items were more common among women with a current mental disorder, except for unstable housing and previous hospital admission of a first-degree relative for mental disorders.

**Table 2:** **Prevalence of characteristics, psychosocial and psychiatric triage items according to the presence of mental disorders** ^**1**^

	**Total (n = 330)**		**Women with mental disorder (n =131)**			**Women without mental disorder (n = 199)**			**Total (n = 330)**		**Women with mental disorder (n =131)**		**Women without mental disorder (n = 199)**	
*Socio-demographic characteristics*	*n*	*(%)*	*n*	*(%)*		*n*	*(%)*	*Life event item*	*n*	*(%)*	*n*	*(%)*	*n*	*(%)*
Maternal age (years)^2^	31	(27 - 35)	32	(29 - 35)		31	(27 - 34)	Sexual or physical abuse^4^	49	(15)	43	(33)	6	(3)
Non-Western ethnicity	121	(37)	58	(44)		63	(32)	*Substance use items*	*n*	*(%)*	*n*	*(%)*	*n*	*(%)*
								Smoking during pregnancy^5^						
Socio economic status^3^								No	212	(64)	76	(58)	136	(68)
< 20^th^ percentile	175	(53)	70	(53)		105	(53)	Yes, untill pregnancy was known	66	(20)	25	(19)	41	(21)
20^th^ - 20^th^ percentile	119	(36)	43	(33)		76	(38)	Yes, still	52	(16)	30	(23)	22	(11)
≥ 80^th^ percentile	36	(11)	18	(14)		18	(9)							
								Alcohol consumption during pregnancy^6^						
Educational level								No	151	(46)	59	(45)	92	(46)
low	44	(13)	20	(15)		24	(12)	Yes, untill pregnancy was known	167	(51)	63	(48)	104	(52)
moderate	168	(51)	75	(57)		93	(47)	Yes, still	12	(4)	9	(7)	3	(2)
high	118	(36)	36	(27)		82	(41)							
								Illicit drug use during pregnancy						
Single status	15	(5)	10	(8)		5	(3)	No	308	(93)	116	(89)	192	(96)
								Yes, untill pregnancy was known	19	(6)	13	(10)	6	(3)
*Obstetric characteristics*	*n*	*(%)*	*n*	*(%)*		*n*	*(%)*	Yes, still	3	(1)	2	(2)	1	(1)
Gestational age (weeks)^2^	24	(14 - 31)	23	(16 - 32)		24	(14 - 31)							
								*Psychiatric items*	*n*	*(%)*	*n*	*(%)*	*n*	*(%)*
Gravidity								Hospital admission for mental disorder ever (woman herself)	35	(11)	24	(18)	11	(6)
1	99	(30)	41	(31)		58	(29)							
2	113	(34)	36	(27)		77	(39)							
3	50	(15)	22	(17)		28	(14)	Hospital admission of a first-degree relative for mental disorder ever	44	(13)	21	(16)	23	(12)
≥4	68	(21)	32	(24)		36	(18)							
Parity								Psychotropic medication use ever	117	(35)	83	(63)	34	(17)
0	152	(46)	65	(50)		87	(44)							
1	110	(33)	35	(27)		75	(38)	Professional psychiatric treatment ever	175	(53)	105	(80)	70	(35)
2	43	(13)	19	(15)		24	(12)							
3	16	(5)	8	(6)		8	(4)	Traumatic experience ever	104	(32)	72	(55)	32	(16)
≥4	9	(3)	4	(3)		5	(3)							
								Feelings of a depressed mood ever	169	(51)	109	(83)	60	(30)
*Psychosocial items*	*n*	*(%)*	*n*	*(%)*		*n*	*(%)*							
Insufficient social support	50	(15)	34	(26)		16	(8)	Panic attac ever	130	(39)	88	(67)	42	(21)
Relational problems	47	(14)	38	(29)		9	(5)	Current psychiatric symptoms	80	(24)	70	(53)	10	(5)
Financial debts	68	(21)	43	(33)		25	(13)	Current severe depressive or anxious symptoms	75	(23)	62	(47)	13	(7)
Unstable housing	15	(5)	10	(9)		5	(3)							
								Current severe fear of childbirth	70	(21)	45	(34)	30	(15)
Unplanned pregnancy	133	(40)	68	(52)		65	(33)							
								Edinburgh Depression Scale score^2^	3	(1 - 10)	12	(5 - 17)	2	(0 - 3)
Unwanted pregnancy	30	(9)	20	(15)		10	(5)							

^*1*^ *Diagnosis based on Structured Clinicial Interview of DSM-IV disorders axis I and II (SCID I and SCID II) by a certified professional.*

^*2*^ *Data given as median (Q1;Q3).*

^*3*^ *Based on a z-score for socio-economic status nationally available at the website of the Central Bureau of Statistics (* *http://statline.cbs.nl/StatWeb/dome/default.aspx* *).*

^*4*^ *Defined as current sexual/physical abuse, or still suffering from a past history of sexual/physical abuse.*

^*5*^ *Defined as smoking at least one cigarette a day.*

^*6*^ *Defined as consuming at least one glass of alcohol a week.*

*All items were part of the Mind2Care screen-and-advice instrument, except for: professional psychiatric treatment ever, traumatic experience ever, feelings of a depressed mood ever, panic attack ever, current psychiatric symptoms, current severe depressiv.*

Table 3 shows the multivariate logistic regression results for the prediction of mental disorders. All models appeared to be statistically sufficiently valid, with an increasing goodness of fit depending on the number of items included, as we would expect.

**Table 3:** **Multivariate logistic regression analysis for four triage models for mental disorders during pregnancy**

	**Model 1**		**Model 2**		**Model 3**		**Model 4**	
*Psychiatric items (short set)* ^*2*^	*OR*	*(95% CI)*						
Hospital admission for mental disorder ever (women herself)								
No (REF)	1							
Yes	1.23	(0.52 - 2.89)						
Hospital admission of a first-degree relative for mental disorder ever								
No (REF)	1							
Yes	1.05	(0.50 - 2.19)						
Psychotropic medication use ever								
No (REF)	1							
Yes	7.96	(4.59 - 13.80)						
*Psychiatric items (intermediate set)* ^*3*^								
			*OR*	*(95% CI)*	*OR*	*(95% CI)*	*OR*	*(95% CI)*
Traumatic experience ever								
No (REF)			1		1		1	
Yes			3.00	(1.58 - 5.70)**	2.78	(1.36 - 5.64)**	1.89	(0.91 - 3.90)
Feelings of a depressed mood ever								
No (REF)			1		1		1	
Yes			3.57	(1.83 - 6.96)**	2.31	(1.10 - 4.85)*	3.58	(1.74 - 7.39)**
Panic attac ever			1		1		1	
No (REF)			1		1		1	
Yes			2.59	(1.35 - 4.94)**	2.50	(1.23 - 5.10)* (1.23 - 5.10)	3.57	(1.75 - 7.28)**
Current psychiatric symptoms			1		1		1	
No (REF)			1		1		1	
Yes			5.08	(2.07 - 12.46)**	2.44	(0.89 - 6.68)	4.56	(1.80 - 11.53)**
Current severe depressive or anxious symptoms			1		1		1	
No (REF)			1		1		1	
Yes			2.40	(1.00 - 5.76)	0.73	(0.25 - 2.11)	2.38	(0.95 - 5.98)
*Psychiatric items (extended set)* ^*3*^					*OR*	*(95% CI)*		
Edinburgh Depression Scale score^4^					1.26	(1.16 - 1.37)		
*Psychosocial items (comprehensive set)* ^*3*^							*OR*	*(95% CI)*
Sexual or physical abuse^5^							1	
No (REF)							5.87	(1.88 - 18.27)**
Yes								
Alcohol consumption during pregnancy^6^								
No (REF)							1	
Yes, untill pregnancy was known							1.10	(0.57 - 2.15)
Yes, still							9.13	(1.78 - 46.74)**
Unplanned pregnancy								
No (REF)							1	
Yes							3.00	(1.52 - 5.90)**

^*1*^ *Diagnosis based on Structured Clinicial Interview of DSM-IV disorders axis I and II (SCID I and SCID II).*

^*2*^ *all determinants were entered.*

^*3*^ *stepwise backwards logistic regression, last step of the regression model is reported.*

^*4*^ *Including 10 items.*

^*5*^ *Defined as current sexual/physical abuse, or still suffering from a past history of sexual/physical abuse.*

^*6*^ *Defined as consuming at least one glass of alcohol a week.*

*p < 0.05, **p < 0.01.

*Model 1, short set of 3 items* ^*,*^ *x² = 75.3, p < 0.001. Nagelkerke's R² = 0.276, HL (Hosmer & Lemeshow) = 6.312.*

*Model 2, intermediate set of 5 items, x² = 171.1, p < 0.001. Nagelkerke's R² = 0.547, HL = 1.454.*

*Model 3, intermediate ste + Edinburgh Depression Scale, together: extended set of 15 items, x² = 210.1, p < 0.001. Nagelkerke's R² = 0.637, HL = 6.432.*

*Model 4, intermediate set + 3 psychosocial items, together: comprehensive set of 8 items, x² = 199.9, p < 0.001. Nagelkerke's R² = 0.615, HL = 14.566.*

**Figure 2:**
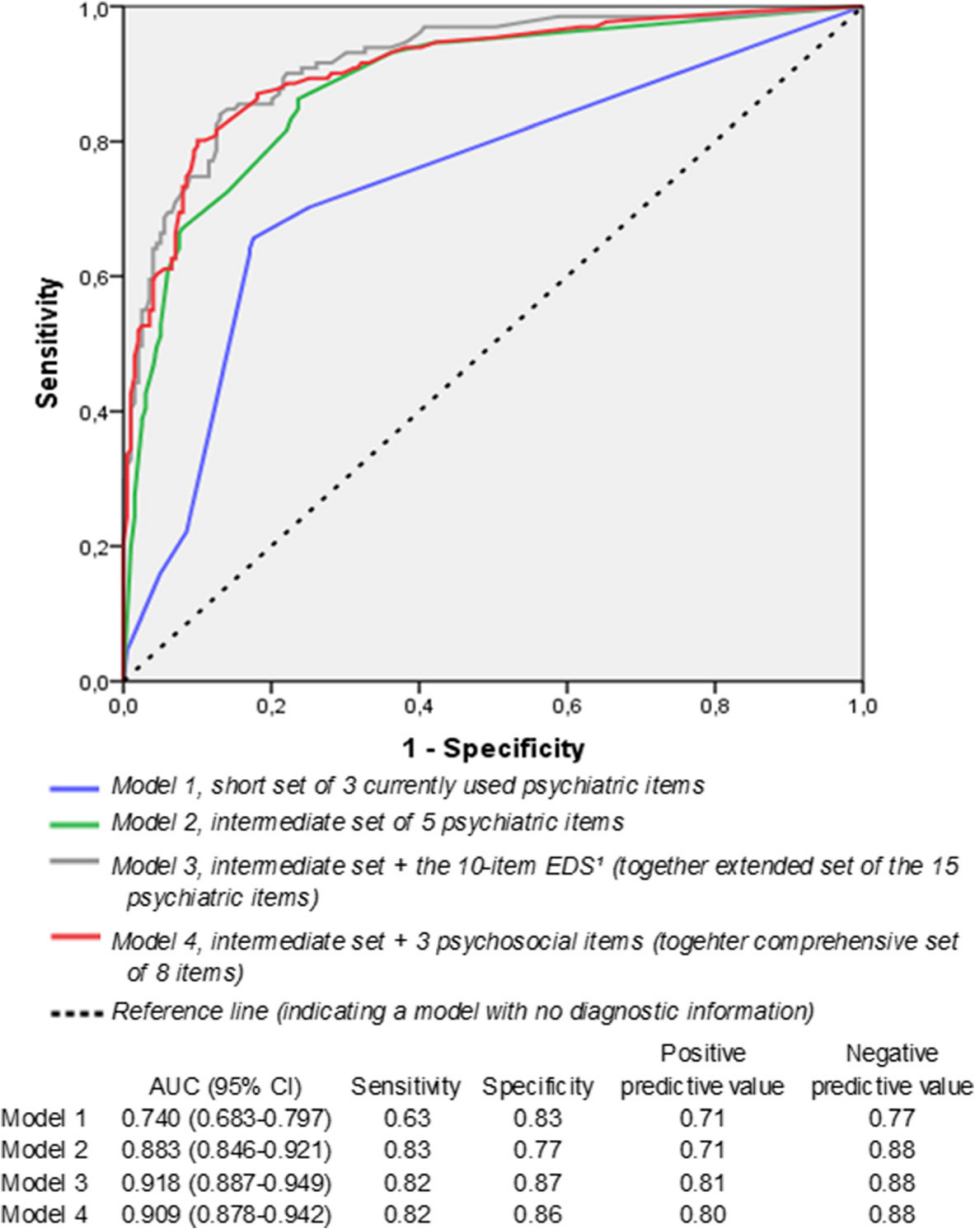
**Area under the ROC curve and discriminant validity of four triage models for mental disorders during pregnancy.**

Model 1 represents the short set of three psychiatric items with a Nagelkerke's R^2^ of 0.276 and Hosmer and Lemeshow’s statistic = 6.312. The AUC was 0.740 (95% CI: 0.683-0.797), representing an acceptable discrimination (Figure 2). The test performance of the model at the cut-off point of 0.4 showed a high specificity and NPV (0.83 and 0.77), yet 48 out of 131 cases were missed and 34 non-cases were falsely identified as a case. Model 2 includes the intermediate set of five psychiatric items for the detection of mental disorders during pregnancy, as nominated through a backwards stepwise regression analysis (see Additional file 2). Nagelkerke's R^2^ was 0.547, Hosmer and Lemeshow was 1.454, and the AUC showed an excellent discrimination of 0.883 (95% CI: 0.846-0.921). Sensitivity and NPV appeared high (0.83 and 0.88). Model 2 missed 22 cases and identified 45 non-cases as cases.

Addition of the 10-item Edinburgh Depression Scale (model 3, extended set of 15 items), increased Nagelkerke's R^2^ with 16% to 0.637, Hosmer and Lemeshow’s statistic to 6.432, and the AUC to 0.918 (95% CI: 0.887-0.949), representing outstanding discrimination. Twenty-three cases were missed and 25 non-cases were falsely identified as a case. The three psychosocial items sexual or physical abuse, alcohol consumption during pregnancy and having an unplanned pregnancy were all identified as significant predictors for mental disorders during pregnancy. The addition of these psychosocial items to the five psychiatric items of model 2, increased Nagelkerke's R^2^ with 12% from 0.547 for model 2 to 0.615 for model 4 (comprehensive set of items). Hosmer and Lemeshow’s statistic increased to 14.566. The AUC slightly increased as well from 0.883 to 0.909, representing an outstanding discrimination, comparable to model 3. Similarly as compared to the addition of the 10-item Edinburgh Depression Scale (model 3), the addition of three psychosocial items did not affect the sensitivity or NPV, but further increased the specificity and PPV to 0.86 and 0.80 respectively. Again, 23 cases were undetected, and 2 more non-cases were falsely identified (n = 27).

Finally, socio-demographic characteristics were added to explore the influence of these characteristics on the performance of the triage models. After applying stepwise backwards regression analysis, none of the characteristics contributed to further improvement of the detection of mental disorders, suggesting indifference of the screening performance tool for socio-demographics.

## Discussion

The burden of a broad spectrum of mental disorders in pregnancy is considerable, and a structured triage is often lacking. Our study showed that the currently used short set of three psychiatric items at least performs acceptably for triage purposes. However, an intermediate set including the five psychiatric items traumatic experience ever, feelings of a depressed mood ever, panic attack ever, current psychiatric symptoms and current severe depressive or anxious symptoms, significantly improves the set’s performance. Further improvement can be achieved by adding the 10-item Edinburgh Depression Scale or at least adding three items on co-morbid conditions: alcohol consumption, physical or sexual abuse, and having an unplanned pregnancy. The addition of these three psychosocial items provides a brief yet broad triage (see Additional file 2).

Many research studies have addressed the validation of questionnaires focusing on one specific topic such as antenatal depression or anxiety [10-14]. To our knowledge, we are the first to screen for the broad spectrum of DSM-IV axis I and axis II disorders during pregnancy instead of focusing on the most prevalent disorders only. As personality disorders contribute to the burden of mental disease, triage creates an opportunity for identifying disorders that are otherwise left unnoticed in the obstetric setting. The rather complex and long lasting treatment of personality disorders is often not a primary aim during pregnancy, however, the provision of some kind of maternal support is desired. In addition, we are the first to systematically investigate the independent predictive role of psychosocial risk factors, following a study of de Graaf et al. [25].

Unlike previous studies, which mostly assess psychiatric diagnoses in screen positive participants [26-28], we obtained psychiatric diagnosis for both low and high-risk participants. This validity check across all participants provides the best information on the test performance of the triage models. At this stage a thorough verification procedure, yielding false negative and false positive rates of the screening, justifies the effort of a psychiatric assessment of all participants.

The purpose of this study was to validate a set of items for the triage of a broad spectrum of mental disorders during the antenatal phase. Clinical triage requires the combination of a high sensitivity, specificity, positive and negative predictive value. The emphasis is on high sensitivity and high negative predictive value (resulting in low rates of missed cases), assuming that false-positive women are identified during an intentional subsequent confirmation by a psychiatric professional. Despite the excellent discrimination of the triage models, 23 women with a current mental disorder were missed. These women are likely to have limited insight into their illness, because all 23 responded negatively to the question about having current psychiatric symptoms. Interestingly three out of five women with a current eating disorder, and two out of eight women with a current psychotic disorder were missed by the triage model, indicating a low sensitivity for these types of disorders. Seven out of the 23 missed cases included first onset of psychiatric disorders in women of moderate to high education from a Western origin, without a psychiatric history and without any psychosocial stressors. Two of these women reported fetal loss or previous miscarriages as reasons for their anxiety disorder. This stresses the importance of special awareness of the psychiatric consequence of previous adverse pregnancy outcomes.

This study was subject to several limitations. Firstly, psychiatric diagnosis addressed a current state of mental disorders and not a future state throughout pregnancy or postpartum. Repeated assessments during pregnancy and postpartum would provide valuable information on the onset of mental disorder during later pregnancy and after delivery. Nevertheless, this study included pregnant women with a mixture of gestational ages, representing the whole antenatal period. As postpartum mental disorders often already start during pregnancy, we focused on the antenatal period only. Secondly, the response rate was relatively low (38%). This was possibly due to the duration of the interview, as women were informed on the approximate length of the interview prior to the study. Baseline characteristics of responders and non-responders were comparable, except for ethnicity. Non-responders were more often of non-Western ethnicity (46% versus 37%, p = 0.008). As women of non-Western ethnicity more often had psychiatric disorders in this study, this could have led to the selection of a healthier population.

## Conclusions

The findings in this study led to an important recommendation. For a brief triage and a subsequent referral to psychiatric care or provision of support for women with mental disorders during pregnancy, the implementation of a comprehensive set of at least five psychiatric triage items is warranted. As the addition of three psychosocial items significantly improves the performance of the triage tool at low cost, we advocate the implementation of this 8-item comprehensive set of items in routine obstetric care (see Additional file 2). Nevertheless, triage alone is not enough. All identified women following triage need a psychiatric consultation for the confirmation of the psychiatric disorder and subsequent interventions.

## Additional files

Additional file 1:
**Ten potential psychiatric items for the triage of mental disorders of the DSM-IV axis I and II during pregnancy.** Description: Additional file 1 includes an overview of the ten potential psychiatric items to be used for the triage of mental disorders during pregnancy. Additional file 2:
**The comprehensive set of five psychiatric items and three psychosocial items to be implemented in routine obstetric care for the triage of mental disorders of the DSM-IV axis I and II.** Description: Additional file 2 includes an overview of the comprehensive set of psychiatric and psychosocial items to be used for the triage of mental disorders in routine obstetric care.

## Abbreviations

AUC: Area under the curve
EDS: Edinburgh depression scale
NPV: Negative predictive value
PPV: Positive predictive value
ROC: Receiver operator characteristics
SCID: Structured clinical interview of the DSM-IV disorders
